# The psychological health and associated factors of men who have sex with men in China: A cross-sectional survey

**DOI:** 10.1371/journal.pone.0197481

**Published:** 2018-05-29

**Authors:** Jie Liu, Zhe Yi, Yang Zhao, Bo Qu, Yaxin Zhu

**Affiliations:** 1 Department of Health Statistics, School of public health, China Medical University, Shenyang, Liaoning Province, P.R. China; 2 Department of Prothodontics, School of Stomatology, China Medical University, Shenyang, Liaoning Province, People’s Republic of China; Public Library of Science, UNITED KINGDOM

## Abstract

**Objectives:**

The psychological health of men who have sex with men (MSM) has received increased attention in recent years. We thus investigated the psychological status and associated factors among MSM in China.

**Methods:**

A cross-sectional survey of 248 MSM was conducted from April to September 2015 using Symptom Checklist 90 (SCL-90) in Huludao and Zhengzhou, China. Statistical analyses utilized SPSS version 19.0 for Windows.

**Results:**

All Cronbach’s α coefficients of the SCL-90 subscales exceeded 0.7, suggesting acceptable reliability. The coefficient range of the collective validity for all the subscales was >0.4. For the divisional validity, each item correlated better with the hypothetical subscale than with other subscales. Collective validity and divisional validity were both acceptable. The four most frequent types of psychological distress among MSM were depression, obsessive-compulsive behavior, interpersonal sensitivity, and anxiety. Results of the univariate analysis revealed that the following groups had significantly higher SCL-90 scores (P < 0.05): peasantry, married MSM, respondents who reported condomless anal intercourse and a greater number of male partners, and respondents who had not undergone psychosocial counseling and whose family or friends did not know about their sexual identity. In a multivariate logistic regression model, the following parameters were independently associated with higher SCL-90 scores: being married (AOR [adjusted odds ratio] = 3.19; 95% CI [confidence interval]: 1.96 to 5.93), condomless anal intercourse (AOR = 1.16; 95% CI: 1.02 to 1.31), number of male partners (AOR = 1.66 and 1.81; 95% CI: 1.08 to 2.34 and 1.32 to 2.69), family or friends not knowing about sexual identity (AOR = 2.13; 95% CI: 1.17 to 4.92), and lack of psychosocial counseling (AOR = 2.09; 95% CI: 1.06 to 4.09).

**Conclusions:**

Our results indicate that psychological health problems among MSM in China are of concern. It is thus necessary to strengthen intervention efforts, with more emphasis on intervention programs to improve psychological health among Chinese MSM.

## Introduction

Studies conducted in India and the Netherlands have revealed that men who have sex with men (MSM), in contrast with their heterosexual counterparts, have poorer mental health and experience more mental distress [1,2]. MSM in the United States report significantly higher rates of lifetime mood or anxiety disorder and have been identified as a higher risk group for depression compared with the general population [3,4]. A growing body of literature from China and France suggests that HIV risk among MSM increases when an individual reports having a relatively greater number of psychological problems [5–8]. Psychological health issues may thus contribute to the propensity for MSM to engage in risky sexual behavior and may affect the degree to which they might benefit from HIV prevention programs [5–8]. Another study from the United States suggests that psychological problems among MSM are highly associated with drug use and risky sexual behavior [9]. Therefore, MSM with psychiatric symptoms might be at greater risk for HIV infection owing to a greater prevalence of sexual relations, multiple partners, and sexual abuse in the United States [10,11].

MSM have long since constituted a high-risk population for HIV infection and have recently become one of the target populations for preventing HIV transmission in China [12–14]. Several studies have reported depression, anxiety, sexual abuse, and other psychological syndemics among MSM in China [5,15–16], where homosexual relationships are not legal and homosexuality is stigmatized in the general public [17]. A study of MSM conducted in nine large Chinese cities found that 44–60% of the respondents felt that their life was substantially negatively affected by their sexual orientation [5]. Compared with heterosexual males, MSM are particularly vulnerable to psychiatric disorders [18]. A study of MSM conducted in four cities of northeast China suggested that Chinese MSM have significantly elevated prevalence and comorbidity of psychiatric disorders compared with heterosexual males [15]. A national survey revealed that 34.5% of Chinese homosexual males had attempted or committed suicide [19].

Another study revealed that higher levels of psychological distress are independently associated with older age, alcohol use, poor self-reported quality of life, and reduced condom use at last sexual encounter [20]. Discrimination, stigma, and socially related stress are often cited as potential contributing factors for the observed elevated psychological distress experienced by MSM [21,22]. Previous literature has also indicated that the prevalent stigma and discrimination against homosexuals and the resulting associations with depression can negatively influence quality of life and ability to make healthy decisions, including decisions regarding HIV preventive behaviors or initiation and adherence to HIV treatment [23–27]. MSM who reported a relative lack of social support and engaged in risky behaviors were more likely to have psychological problems [20,28]. Owing to the substantial mental health implications, more attention should be paid to the psychosocial health problems among the MSM population. However, very few studies have assessed the psychological health status of this population in China.

The Symptom Checklist 90 (SCL-90) has been one of the most reliable psychopathological testing tools for clinical populations worldwide and is becoming one of the most popular measures with which to assess psychiatric distress in diverse cultures worldwide. SCL-90 is a self-reporting tool that has proven reliable and valid for psychological evaluation [29,30], including its use in HIV/AIDS studies [31–33]. The Chinese version of the SCL-90 has also proven reliable and valid in studies of the general population in China [33,34].

The primary objective of this study was to assess the psychological health status and explore associated factors among MSM in China using SCL-90. We hypothesized that there might be high rates of mental health problems among MSM and that many factors likely contribute to the psychological problems of individuals in the MSM population. The findings help us understand the psychological status of MSM and the possible influencing factors that will inform the development of prevention strategies.

## Materials and methods

### Respondents and procedures

A cross-sectional study was conducted from April to September 2015 in two Chinese cities, Zhengzhou and Huludao. The initial sample size (N = 203) was calculated according to the formula *n* = 100 × (1 − *p*) / *p*. It has been reported that 33.0–46.1% of MSM experience symptoms of depression or anxiety disorders in China [6,8,31]. To ensure an adequate sample size, the lowest prevalence rate (33%) was used as the *p* value. The sample size was increased to 248 to take into account any lack of response from the initial 203 individuals. In total, 226 MSM completed the survey (response rate: 91.1%). Respondents were recruited from an internet advertisement, bars, and saunas. The respondents underwent a face-to-face explanation of the plan and intent of our study prior to taking the standardized questionnaire The inclusion criteria were that respondents: (1) were 18 years of age or older, (2) had had sex with a man in the preceding 6 months, and (3) were without cognitive impairment. All questionnaires were self-administered in a private room. Only participants who gave written informed consent were enrolled in the study. The study protocol was approved by the bioethics advisory commission of China Medical University.

### Questionnaires

The questionnaire was divided into three parts. The first part included socioeconomic characteristics such as age, vocation, marital status, education level, monthly income, sexual orientation, and family or friend awareness of sexual orientation. The second part concerned sexual-related behaviors and use of social services. Respondents were asked whether their first sex partner was male or female, the number of male partners in the preceding 6 months, whether they had engaged in unprotected anal intercourse (UAI), and whether they had sex with a female in the preceding 6 months. UAI was defined as having had at least one incident of condomless anal intercourse with any male partner in the preceding 6 months. Questions pertaining to use of social services included receiving HIV/syphilis testing, receiving AIDS information materials, receiving peer education, receiving AIDS counseling, and receiving psychosocial counseling. The third part was a Chinese version of SCL-90, which includes 90 items related to psychological health of an individual and contains nine subscales (somatization, obsessive-compulsive, interpersonal sensitivity, depression, anxiety, anger/hostility, phobic anxiety, paranoid ideation and psychoticism) and an additional scale that assesses disturbances in appetite and sleep. Respondents answered each question using a five-point scale (1 = ‘not at all’, 2 = ‘a little bit’, 3 = ‘moderately’, 4 = ‘quite a bit’, and 5 = ‘extremely’) as relevant to symptoms experienced in the preceding 7 days. The responses to items relevant to each subscale were averaged to give a subscale score and, additionally, responses to all items were summed to give a total score. Any subscale score of >2.0 or a total score of >160 was considered a threshold for identifying individuals who require further evaluation [35,36]. Higher scores on the SCL-90 represent more serious psychological distress [35,36].

### Statistical analysis

Cronbach’s α coefficient was used to assess the internal consistency of the SCL-90 items, and validity was assessed by collective validity and divisional validity. A t-test and one-way analysis of variance were used to identify factors related to psychological status. Logistic regression analyses were performed to identify factors associated with SCL-90 scores. Variables with a P-value < 0.10 for univariate results were considered eligible for the multivariate analysis. The significance level was fixed at α = 0.05. All statistical analyses were carried out using SPSS^®^ version 19.0 for Windows^®^ (SPSS Inc., Chicago, IL, USA).

## Results

### Evaluation of reliability and validity

The degree of internal uniformity among the items of the SCL-90 questionnaire was expressed by Cronbach’s α coefficient. The overall Cronbach’s α coefficient was 0.844, which ranged from 0.810 (the depression dimension) to 0.959 (the phobic anxiety dimension). All Cronbach’s α coefficients of the subscales exceeded 0.7 and met the requirement for group comparison, suggesting acceptable reliability. The coefficient range of the collective validity for all the scales was >0.4. For the divisional validity, items have higher correlation with their hypothesized scales than with scales used to measure other concepts. Collective validity and divisional validity were good. Table 1 presents the results for collective validity and divisional validity of the SCL-90.

**Table 1:** Collective validity and divisional validity of the SCL-90 questionnaire.

Scale	Number^a^	Coefficient range		Collective validity		Divisional validity	
		Collective validity*	Divisional validity*	Success/total ^b^	Success rate (%)	Success/total ^c^	Success rate (%)
Somatization	12	0.545–0.676	0.093–0.536	12/12	100	120/120	100
Obsessive-compulsive	10	0.679–0.756	0.141–0.497	10/10	100	100/100	100
Interpersonal sensitivity	9	0.703–0.894	0.139–0.341	9/9	100	90/90	100
Depression	13	0.734–0.908	0.085–0.479	13/13	100	130/130	100
Anxiety	10	0.704–0.897	0.154–0.517	10/10	100	100/100	100
Anger/hostility	6	0.505–0.721	0.116–0.442	6/6	100	60/60	100
Phobic anxiety	7	0.654–0.802	0.128–0.494	7/7	100	70/70	100
Paranoid-ideation	6	0.564–0.655	0.186–0.444	6/6	100	60/60	100
Psychoticism	10	0.571–0.806	0.212–0.375	10/10	100	100/100	100
Other	7	0.487–0.788	0.227–0.425	7/7	100	70/70	100

^a^:Items per scale.

^b^:Number of correlations between items and hypothesised scale corrected for overlap > 0.40/total number of collective validity tests.

^c^:Number of correlations significantly higher/total number of divisional validity tests.

* For collective validity, success means correlations between items and hypothesized scale corrected for overlap > 0.40, and for divisional validity success means items in a dimension were more highly correlated with their hypothesized dimension than with other dimensions.

### Sociodemographic characteristics

A total of 226 MSM completed the questionnaire. The age of respondents was 28.3 ± 8.6 years (range 18–68 years), and 94 (41.6%) self-identified as bisexual, 166 (73.5%) said they were single, and 60 (26.5%) were married. Education level was reported as follows: junior high school or lower (17.2%), senior high school (30.5%), and college or above (52.3%). Monthly income was categorized as: 1000–2000 Yuan (30.5%), 2001–3000 Yuan (42.5%), 3001–5000 Yuan (19.5%), and >5000 Yuan (7.5%). Respondent occupations were as follows: factory workers (18.2%), peasants (21.7%), administrators (21.2%), service personnel (32.7%), and students (6.2%). Among all respondents, 11.9% had disclosed their sexual orientation to family or friends.

### Sexual behaviors and social service utilization

Among respondents, 75.2% reported that their first sex partner was male, 35.8% reported having only one male partner in the preceding 6 months, and 47.3% had engaged in condomless anal intercourse in the preceding 6 months. Additionally, 21.2% reported having had sex with a woman in the preceding 6 months. The proportions receiving HIV/syphilis testing and informational materials were 61.1% and 78.8%, respectively. A total of 62.8%, 72.6%, and 9.7% of the respondents received peer education, AIDS counseling, and psychosocial counseling, respectively.

### Assessment of psychosocial health

Among our participants, the highest score was for depression (mean ± SD: 1.64 ± 0.70) and the lowest score was for somatization (mean ± SD: 1.39 ± 0.57). The total score for 46 of the participants was >160, suggesting psychological distress. The four domains having the highest scores were depression (46/226), obsessive-compulsive (44/226), interpersonal sensitivity (35/226), and anxiety (30/226). Compared with the Chinese norm [37], our MSM population had higher scores except for somatization and interpersonal sensitivity (P < 0.05; Table 2).

**Table 2:** Scores for nine dimensions of SCL-90 among MSM and comparisons with Chinese norm.

	MSM(n = 226, mean±SD)	Chinese norms^a^(n = 7273, mean±SD)	t value
Somatization	1.39±0.57	1.40±0.40	0.364
Obsessive-compulsive	1.62±0.65	1.49±0.54	3.540*
Interpersonal sensitivity	1.50±0.60	1.45±0.52	1.417
Depression	1.64±0.70	1.42±0.49	6.546*
Anxiety	1.48±0.66	1.31±0.42	5.865*
Anger/hostility	1.47±0.64	1.37±0.50	2.933*
Phobic anxiety	1.49±0.70	1.25±0.40	6.670*
Paranoid-ideation	1.52±0.70	1.35±0.49	5.058*
Psychoticism	1.47±0.64	1.22±0.37	9.717*

SCL-90, Symptom Checklist 90; MSM, men who have sex with men.

*P < 0.05.

^a^: The data of Chinese norms in the table originate from a previously published article[37]

### SCL-90 scores stratified by sociodemographic characteristics

The oldest group had a significantly higher mean score for the somatization subscale (1.68 ± 0.68, P < 0.05), and the youngest group had a significantly higher mean score for the phobic anxiety subscale (4.04 ± 0.96, P < 0.05). The peasantry had significantly higher scores for mean total score of SCL-90 (173.07 ± 63.52, P < 0.05), somatization (1.89 ± 0.71, P < 0.05), depression (2.02 ± 0.74, P < 0.05), paranoid-ideation (2.21 ± 0.50, P < 0.05), and psychoticism (2.08 ± 0.89, P < 0.05). The married MSM had significantly higher scores for mean total score of SCL-90 (171.92 ± 64.20, P < 0.05), obsessive-compulsive (2.02 ± 0.69, P < 0.05), interpersonal sensitivity (1.94 ± 0.71, P < 0.05), depression (2.38 ± 0.77, P < 0.05), anxiety (1.87 ± 0.89, P < 0.05), and psychoticism (1.98 ± 0.78, P < 0.05). The respondents with junior high school or lower education level had higher scores for mean phobic anxiety (2.26 ± 0.81, P < 0.05) and paranoid-ideation (1.82 ± 0.64, P < 0.05). Respondents who disclosed their sexual orientation to family or friends had lower scores for SCL-90 (120.06 ± 34.19, P < 0.05), depression (1.31 ± 0.55, P < 0.05), and anxiety (1.29 ± 0.38, P < 0.05). Table 3 presents the SCL-90 total scores stratified by sociodemographic characteristics.

**Table 3:** SCL-90 total scores stratified by sociodemographic characteristics.

Item	Number	Percentage (%)	Total score (mean±SD)
**Age**			
≤20	31	13.7	150.08±52.50
21–30	114	50.5	134.78±48.10
31–40	45	19.9	132.75±49.15
≥41	36	15.9	149.73±50.21
**Vocation**			
Worker	41	18.2	136.39±50.53*
Peasantry	49	21.7	173.07±63.52
Administrator	48	21.2	134.80±53.73
Service Personnel	74	32.7	136.97±58.03
Student	14	6.2	130.15±38.44
**Marital status**			
Single	166	73.5	134.78±48.95*
Married	60	26.5	171.92±64.20
**Education level**			
Junior high school or lower	39	17.2	149.77±54.31
Senior high school	69	30.5	129.06±38.22
College or above	118	52.3	137.26±52.61
**Monthly income (Yuan)**			
1000–2000	69	30.5	137.91±49.47
2001–3000	96	42.5	144.04±56.55
3001–5000	44	19.5	124.34±47.08
>5000	17	7.5	125.18±26.48
**Family or friends know about sexual identity**			
Yes	27	11.9	120.06±34.19*
No	199	88.1	142.75±57.67

SCL-90, Symptom Checklist 90.

*P < 0.05

### SCL-90 scores stratified by sexual behaviors and utilization of social services

No significant differences were found among different groups in terms of first sex partner (P > 0.05). Respondents with a greater number of male partners (≥4) had higher scores for somatization (1.82 ± 0.74, P < 0.05), obsessive-compulsive (2.04 ± 0.97, P < 0.05), interpersonal sensitivity (1.91 ± 0.78, P < 0.05), depression (2.03 ± 0.96, P < 0.05), anxiety (1.91 ± 0.12, P < 0.05), paranoid-ideation (1.91 ± 0.21, P < 0.05), psychoticism (1.91 ± 0.51, P < 0.05), and other (1.81 ± 0.73, P < 0.05). Respondents who engaged in condomless anal intercourse in the preceding 6 months had higher scores for subscales: total score (151.42 ± 44.17, P < 0.05), somatization (1.51 ± 0.57, P < 0.05), obsessive-compulsive (1.77 ± 0.65, P < 0.05), interpersonal sensitivity (1.72 ± 0.66, P < 0.05), depression (1.83 ± 0.62, P < 0.05), anxiety (1.62 ± 0.58, P < 0.05), anger/hostility (1.61 ± 0.56, P < 0.05), phobic anxiety (1.77 ± 0.46, P < 0.05), paranoid-ideation (1.73 ± 0.44, P < 0.05), and psychoticism (1.62 ± 0.78, P < 0.05). Respondents who had sex with a female in the preceding 6 months had a lower mean interpersonal sensitivity score (1.41 ± 0.49, P < 0.05). Table 4 presents the SCL-90 total scores stratified sexual behavior characteristics.

**Table 4:** SCL-90 total scores stratified by sexual behaviors.

Item	Number	Percentage (%)	Total score (mean±SD)
**First sex partner**			
Male	170	75.2	136.95±51.62
Female	56	24.8	136.80±50.57
**Number of male partners in the preceding 6 months**			
1	81	35.8	133.57±50.58*
2–3	116	51.4	133.60±45.21
≥4	29	12.8	171.15±66.90
**Condomless anal intercourse**			
Yes	107	47.3	151.42±44.17*
No	119	52.7	124.55±39.40
**Had sex with female in the preceding 6 months**			
Yes	48	21.2	125.1±42.18
No	178	78.8	140.1±53.09

SCL-90, Symptom Checklist 90.

*P < 0.05

Respondents who received HIV/syphilis testing had lower scores for somatization (1.28 ± 0.49, P < 0.05), anxiety (1.36 ± 0.53, P < 0.05), and hostility (1.36 ± 0.57, P < 0.05). Respondents who received AIDS counseling had lower scores for somatization (1.22 ± 0.36, P < 0.05), anxiety (1.34 ± 0.45, P < 0.05), and hostility (1.33 ± 0.47, P < 0.05). Respondents who receiving psychological counseling had lower scores for the subscales: somatization (1.12 ± 0.26, P < 0.05), obsessive-compulsive (1.25 ± 0.40, P < 0.05), interpersonal sensitivity (1.30 ± 0.51, P < 0.05), depression (1.32 ± 0.46, P < 0.05), anxiety (1.21 ± 0.42, P < 0.05), anger/hostility (1.21 ± 0.37, P < 0.05), phobic anxiety (1.17 ± 0.37, P < 0.05), psychoticism (1.19 ± 0.36, P < 0.05), and other (1.18 ± 034, P < 0.05). Table 5 presents results concerning the utilization of social services.

**Table 5:** SCL-90 total scores stratified by the utilization of social services.

Item	Number	Percentage (%)	Total score (mean±SD)
**Receipt of HIV/syphilis testing**			
Yes	138	61.1	129.39±44.76
No	88	38.9	141.72±54.61
**Receipt of AIDS information materials**			
Yes	178	78.8	134.40±40.37
No	48	21.2	137.60±53.89
**Receipt of peer education**			
Yes	142	62.8	133.86±47.31
No	84	37.2	142.08±57.23
**Receipt of AIDS counseling**			
Yes	164	72.6	129.89±37.82
No	62	27.4	139.57±55.36
**Receipt of psychological counseling**			
Yes	22	9.7	110.57±31.69*
No	204	90.3	139.61±52.15

SCL-90, Symptom Checklist 90.

*P < 0.05

### Factors associated with psychological levels among MSM as assessed with multivariate analysis

In the univariate analysis, SCL-90 total scores were significantly associated with the various vocations, marital status, number of male partners, condomless anal intercourse in the preceding 6 months, family or friends not knowing about sexual identity, and no receipt of psychosocial counseling (P < 0.05). To adjust significant background variables, logistic regression was used to identify factors associated with psychological levels for the total score of SCL-90 (<160 vs. ≥160). Significant factors included in the final model were as follows: married (AOR [adjusted odds ratio] = 3.19; 95% CI [confidence interval]: 1.96 to 5.93), condomless anal intercourse (AOR = 1.16; 95% CI: 1.02 to 1.31), number of male partners (AOR = 1.66 and 1.81; 95% CI: 1.08 to 2.34 and 1.32 to 2.69), family or friends not knowing about sexual identity (AOR = 2.13; 95% CI: 1.17 to 4.92), and no receipt of psychosocial counseling (AOR = 2.09; 95% CI: 1.06 to 4.09). Table 6 presents the results of the logistic regression analysis.

**Table 6:** Factors associated with psychological levels among MSM in multivariable logistic regression model.

Variables in the final model	psychological levels(< 160 vs.≥160)	
	AOR	95% CI
**Marital status**		
single	1	
married	3.19	1.96–5.93*
**Condomless anal intercourse**		
No	1	
Yes	1.16	1.02–1.31*
**Number of male partners**		
1	1	
2–3	1.66	1.08–2.34*
≥4	1.81	1.32–2.69*
**Family or friends know about sexual identity**		
Yes	1	
No	2.13	1.17–4.92*
**Receipt of psychosocial counseling**		
Yes	1	
No	2.09	1.06–4.09*

SCL-90, Symptom Checklist 90; MSM, men who have sex with men; AOR, adjusted odds ratio; CI, confidence interval.

*P < 0.05

## Discussion

In this study, the psychological health of Chinese MSM was assessed based on SCL-90. The SCL-90 has proven reliable and valid for our purpose. The four most frequent types of psychological distress were depression, obsessive-compulsive behavior, interpersonal sensitivity, and anxiety. Both the univariate analysis and the multivariate analysis showed that being married, condomless anal intercourse, the number of male partners, family or friends not knowing about sexual identity, and no receipt of psychological counseling were significantly associated with the SCL-90 score.

MSM are disproportionally affected by mental health problems, a finding that has been consistent across research studies and has received increasing attention [38–40]. In the western-based literature, homosexual and bisexual men are reported to have higher risk for mental health problems such as depression, anxiety, substance abuse, and suicidal ideation as compared with the general population [3,4, 41]. A meta-analysis of studies of lesbians, gay men, and bisexuals found a 2-fold elevated risk of experiencing a psychiatric disorder lasting up to 1 year (OR [odds ratio] = 2.03, 95% CI = 1.68–2.46) or a lifetime (OR = 2.41, 95% CI = 1.91–3.02) [42]. A systematic review revealed that depression, anxiety, and alcohol and substance misuse were at least 1.5-times more common in lesbian, gay, and bisexual people [43]. A study conducted with MSM in India suggested that 29% of 150 MSM in Mumbai had experienced major depression and 24% experienced anxiety at some point in their life [1]. To date, little is known regarding the mental and psychological characteristics of MSM in China. More than 20% of the MSM population in our investigation had a SCL-90 score of >160. Thus, our study indicates that the psychological status of MSM may be cause for concern in China. Therefore, MSM in China represent a diverse population with a broad range of psychological and health needs. Our findings provide information for future public health programs for the prevention and intervention of AIDS.

Social support has also been related to physical and mental health in the general populations worldwide and also for individuals with HIV [33,44,45]. It has been reported that effective social support can reduce substance abuse, promote other positive health behaviors, and improve mental well-being among MSM [46]. In our study, the respondents who had received psychological counseling generally had lower SCL-90 scores and good psychological health, consistent with other research. Counseling to reduce stress has been found to effectively enhance interventions and reduce HIV-related high-risk behaviors among MSM [46,47]. Bastardo and Kimberlin suggested that psychological support is a critical element of HIV care and can help enhance the quality life of patients in developing countries [48]. Psychosocial counseling can also help reduce fear of taking the HIV test and contributes to increased HIV testing among MSM [46]. Although the benefits of psychological counseling are obvious to researchers, only 9.7% of the respondents in our study had received this type of support. Enhancing psychological counseling for Chinese MSM is thus an urgent priority and might be an effective means of improving psychological health.

In our study, family or friends knowing about one’s MSM identity was also associated with a lower SCL-90 total score. A potential explanation for this protective effect is that those who disclosed their sexual orientation to relatives or friends are generally more accepting of their identity, i.e., they may be less likely to experience social pressures and stress related to their sexual orientation. In our study, only 11.9% of MSM had disclosed their sexual orientation to relatives or friends. More interventions could be beneficial to the psychological health of Chinese MSM who do not disclose their orientation.

Houston et al. suggested that a relatively larger number of depressive symptoms is associated with an increased propensity to engage in risky behaviors, e.g., a larger number of sexual partners and frequency of UAI [49]. A study conducted in India also suggested that a relatively larger number of depressive symptoms is associated with having had UAI and a larger number of male partners; for each additional sexual partner, there was an associated 4% increase in the number depressive symptoms [21]. In our study, larger numbers of male partners and condomless anal intercourse were significantly associated with higher SCL-90 scores. We found that those MSM who engaged in condomless anal intercourse had a 1.16-times greater risk of having psychological health problems than those who had not. SCL-90 scores increased with number of male partners, and respondents who had more than four male partners were approximately 2-times more likely to have psychological health problems than those who had one male partner. Our study thus suggests that psychological health is negatively impacted by risky sexual behaviors in accordance with other studies. Stall et al. suggested that psychosocial health problems are associated with exacerbated HIV risk behavior among MSM in the United States [10]. It also has been reported that MSM who have certain psychosocial health problems are more likely to report risky sexual behaviors [50]. Larger numbers of psychosocial health problems are also associated with greater prevalence for high-risk sexual behavior and HIV infection [51]. Studies have suggested that depression and anxiety undermine the intention of individuals to take up protective measures such as condoms, and increased HIV-related sexual risk behavior among MSM correlates with more psychological health problems [4,52]. Therefore, more mental health support services should be provided to the MSM population who practice risky sexual behaviors.

In our study, we found that the psychological status of married MSM was worse than that of unmarried MSM, especially in terms of the obsessive-compulsive behavior and depression, for which the measured values from our questionnaire were greater than the threshold of 2.0. Potential reasons could be that married MSM had experienced both social pressure and family pressure [53]. Because of potential fear of losing social status, feelings of guilt towards family, loneliness, and perceptions of immorality/abnormality, MSM are vulnerable to a variety of psychological difficulties such as serious depression, anxiety, and stress [15,53]. Rui Wang et al. suggested that unmarried MSM have better mental health compared with married MSM [54]. Therefore, more attention needs to be paid to the psychosocial health of married MSM.

Our study revealed that respondents with a junior high school or lower education level had higher SCL-90 total scores, and this was also true of the subscale scores for phobic anxiety and paranoid-ideation. Ning Liu et al. proposed that, in China, higher education level is usually associated with higher social class and economic status [55,56]. Results from studies conducted in Spain and China have shown that people with relatively lower education levels experience more stress and a poorer quality of life [56,57]. Another study concluded that, in China, higher education levels are generally associated with greater knowledge concerning how to deal with pressure [58]. We also found that, compared with other vocations, the MSM peasantry had the highest scores in all domains (P < 0.05), and the total SCL-90 scores were >160, which suggests that members of the MSM peasantry in China have a poor psychological status. In China, the peasantry live in very traditional rural communities, where homosexuality may be less understood/accepted than in urban areas; moreover, there are few entertainment opportunities in such communities [13]. Difficult living conditions and social ignorance may thus contribute to the relatively poor mental status of the MSM peasantry of China.

This study has important limitations. Owing to the social stigma of homosexuality in China and the consequent reclusive nature of the MSM population, potential respondents may have decided not to participate in our study to protect their privacy; indeed, the sample size was relatively small, and the participants were recruited from only two cities in China. Therefore, our respondent group may not be representative of all Chinese MSM. Data were self-reported, and thus social desirability bias may exist. Otherwise, our study used cross-sectional data, which precluded conclusions regarding causality. Despite this limitation, our findings suggest that psychological health problems may be an important area for further research and future intervention among the Chinese MSM population.

## Conclusion

Our results indicate that psychological health problems among MSM in China are of concern. It is thus necessary to strengthen intervention efforts, with more emphasis on intervention programs to improve psychological health among Chinese MSM.
